# Gradient-induced long-range optical pulling force based on photonic band gap

**DOI:** 10.1038/s41377-024-01452-y

**Published:** 2024-04-24

**Authors:** Wenlong Lu, Alexey V. Krasavin, Sheng Lan, Anatoly V. Zayats, Qiaofeng Dai

**Affiliations:** 1 Guangdong Basic Research Center of Excellence for Structure and Fundamental Interactions of Matter, Guangdong Provincial Key Laboratory of Nanophotonic Functional Materials and Devices, School of Information and Optoelectronic Science and Engineering, South China Normal University, Guangzhou, 510006 China; 2 Department of Physics and London Centre for Nanotechnology, King’s College London, London, WC2R 2LS UK

**Keywords:** Optical manipulation and tweezers, Photonic crystals

## Abstract

Optical pulling provides a new degree of freedom in optical manipulation. It is generally believed that long-range optical pulling forces cannot be generated by the gradient of the incident field. Here, we theoretically propose and numerically demonstrate the realization of a long-range optical pulling force stemming from a self-induced gradient field in the manipulated object. In analogy to potential barriers in quantum tunnelling, we use a photonic band gap design in order to obtain the intensity gradients inside a manipulated object placed in a photonic crystal waveguide, thereby achieving a pulling force. Unlike the usual scattering-type optical pulling forces, the proposed gradient-field approach does not require precise elimination of the reflection from the manipulated objects. In particular, the Einstein-Laub formalism is applied to design this unconventional gradient force. The magnitude of the force can be enhanced by a factor of up to 50 at the optical resonance of the manipulated object in the waveguide, making it insensitive to absorption. The developed approach helps to break the limitation of scattering forces to obtain long-range optical pulling for manipulation and sorting of nanoparticles and other nano-objects. The developed principle of using the band gap to obtain a pulling force may also be applied to other types of waves, such as acoustic or water waves, which are important for numerous applications.

## Introduction

Since Ashkin’s pioneering work on optical tweezers^1–3^, optical manipulation has made a remarkable impact in the fields of biology^4,5^, chemistry^6^, quantum physics^7,8^, nanotechnology^9,10^, etc. In these applications, pushing^1^, trapping^3^, lateral shifting^11^, and rotating^9^ forces can be used for complex optical manipulation of nano-objects. Recently, optical pulling has attracted significant attention due to its counter-intuitive nature and interesting underlying physics^12^. The optical pulling force (OPF), capable of pulling an illuminated object towards the light source positioned at a long distance, provides a new degree of freedom in optical manipulation^13^. Optical pulling has unique potential applications in remote backward transportation as well as optical sorting. Expanding the optical pulling concept to waves on water, the pulling force has been suggested for rescuing drowning people and collecting rubbish floating in the sea^14^.

Several approaches were proposed to achieve OPF, including the use of structured beams^13,15,16^, designing objects with exotic parameters^17,18^, structured material surroundings^19–23^, and photophoresis effects^24,25^. Among them, except for the photophoresis effects, almost all the long-range OPFs originate from scattering forces. In this design, OPF is achieved using the law of conservation of linear momentum, with an attempt to increase a forward momentum component of the scattered light, so that the object obtains the reverse recoil momentum.

As a counterpart of the scattering force, a gradient force was anticipated to play a role in the OPF formation^21,26–28^. It was proposed to use a waveguide geometry with either absorption in the waveguide material^26^ or a frequency of the incident light below the cutoff^27,28^ in order to generate an attenuated mode, which results in the light intensity gradient leading to an OPF. However, the OPF generated by such an attenuated mode has drawbacks in practical applications because its penetration distance is very limited^12^. To break this limitation, a different approach to gradient-induced long-range OPF was proposed in ref. ^21^. It was shown that when an object is placed in a region where a self-collimated mode—a unique Bloch mode which can propagate in a uniform photonic crystal (PC) without diffraction—exists, a negative gradient of the light intensity can occur inside the object, which may result in an OPF. However, the origin of the OPF induced by the intensity gradients was not clarified, leading to various misconceptions.

For a point dipole or a Rayleigh particle (r ≪ λ), the well-known gradient force is described by^3,29^

1 $${{\bf{F}}}_{{\rm{grad}}}=\frac{2\pi \alpha }{c{n}_{{\rm{m}}}^{2}}\nabla {I}_{0}$$

where *α* is the polarizability of the sphere, *c* is the speed of light in vacuum, ${n}_{\text{m}}$ is the index of refraction of the surrounding medium and ${I}_{0}$ is the intensity of the light field. As a result, the Rayleigh particle is subjected to a force along the direction of the intensity gradient. It should be emphasized that ${I}_{0}$ here is the intensity of the *incident* field, which is considered to be practically unperturbed by the presence of the particle. For an object with dimensions comparable to or greater than the wavelength, although its presence can significantly perturb the incident field, in many cases the object is still drawn towards the focus by the intensity gradient of the incident field. The most important example is optical tweezers, which use a strongly focused beam to trap objects ranging in size from tens of nanometers to tens of micrometers^30^. This may lead to a wrong notion that the intensity gradient of the incident light is a necessary condition to obtain gradient forces for particles of all sizes. In this case, the particles would always be driven towards the maximum intensity of incident light rather than the light source. This misconception hindered the efforts in obtaining the OPF using gradient forces. However, the particle in reality experiences the *total* local field, and the correct approach to describing gradient forces should rely on the intensity gradient of the *total* local field^31^. This total field is the sum of the incident and the scattered fields $({I}_{\text{total}}=\text{|}{{\bf{E}}}_{0}+{{\bf{E}}}_{\text{scat}}{\text{|}}^{2})$. For example, in a self-collimation effect in PCs, it was found that the OPF can be realized using a negative intensity gradient of the total field inside the object^21^. Nevertheless, a more general way to achieve the negative gradient of field intensity inside the illuminated object is still absent, and a formalism for calculating the gradient forces based on the local total field intensity inside an object needs to be developed. These two factors can provide powerful guidance for designing OPFs in various environments.

The exact approach to find the distribution of electromagnetic force density inside matter is still a controversial topic. The Lorentz (LO) law for electromagnetic force is one of the foundations of classical electrodynamics. One can obtain the LO formulation of the force density distribution by applying the LO law to the density distributions of charge and current^32^. However, this widely accepted formulation was found to violate the universal linear momentum conservation under certain conditions involving magnetic media, and an additional hidden momentum of the electromagnetic field was introduced to explain the imbalance^33^. This hidden momentum has not been observed experimentally, while the explanation of its nature has been widely discussed until now^34,35^. An alternative approach for determining the distribution of electromagnetic force density is based on the Einstein-Laub (E-L) formulation. It complies with the universal conservation laws and does not require the introduction of a hidden momentum^34,36^. Notably, for these two formulations, the total forces exerted by electromagnetic fields on any object are identical when the hidden momentum is included in the Lorentz force on magnetic media^31,37^. Therefore, since many experiments that validate the LO formulation measure the total force acting on an object, these experiments can be also considered as verification of the E-L formulation^38,39^. Further, Minkowski and Abraham also proposed another two formulations that are not entirely identical, assuming that the force only occurs when $\nabla \varepsilon$ or $\nabla \mu$ is nonzero for media without free charges or currents ($\varepsilon$ and $\mu$ being the relative permittivity and relative permeability of the media, respectively)^39,40^. Compared with the Abraham formulation, the E-L formulation has an extra term 1/2 (**P** · **E**) for nonmagnetic media without free charges or currents (**P** and **E** are the polarization density and total electric field, respectively)^39^. The study of the optical force distribution inside matter is an active research topic and the ongoing theoretical discussions and experimental comparisons for various formulations can be found in numerous works^39,41,42^. The aim of this work is to find a suitable approach to guide the design of the OPF and to understand the origin of the optical gradient force.

In this Letter, we implement the E-L formalism to design an OPF. In this formulation, the force density can be clearly expressed in terms of an intensity gradient of the total local field inside objects. We demonstrate that an object placed in a line-defect waveguide in a PC influences the dispersion of the optical guided mode and can produce a spectral band gap, which leads to a decaying profile of the optical intensity envelope inside the object. The interplay between the resulting uncompensated positive and negative intensity gradients inside the object, produces the OPF, as demonstrated by numerical simulations as well as calculations based on the E-L formula. The OPF can also be enhanced by an appropriate design of the PC and the use of an optical resonance in the waveguide transverse direction, excited in the presence of a manipulated object. In such a configuration, high reflection is no longer a prohibiting factor that needs to be eliminated to achieve an OPF. PCs provide a flexible platform for achieving the OPF and any PC with a band gap and a waveguide can be used to realize the OPF.

## Results

The optical force density inside an object (for simplicity, a linear, non-absorbing medium is considered) can be written using the E-L approach as^31^

2 $$\langle {{\bf{f}}}_{{\rm{EL}}}\rangle =\frac{1}{4}{\varepsilon }_{0}[{\varepsilon }_{{\rm{p}}}-1]\nabla ({\bf{E}}\cdot {{\bf{E}}}^{\ast })+\frac{1}{4}{\mu }_{0}[{\mu }_{{\rm{p}}}-1]\nabla ({\bf{H}}\cdot {{\bf{H}}}^{\ast })$$

where $\langle {{\bf{f}}}_{{\rm{EL}}}\rangle$ denotes the time-averaged force density, **E** is the total electric field, **H** is the total magnetic field, ${\varepsilon }_{{\rm{p}}}$ and ${\mu }_{{\rm{p}}}$ are the relative permittivity and relative permeability of the object, respectively. In the following, we will only consider objects without magnetic response for which *µ*_p_ = 1 and the second term in Eq. (2) is absent. The total time-averaged force is then given by

3 $$\langle {{\bf{F}}}_{{\rm{EL}}}\rangle ={\iiint }_{{\rm{object}}}\langle {{\bf{f}}}_{{\rm{EL}}}\rangle dV$$

It is clear from Eqs. (2) and (3) that to achieve the OPF, the negative force caused by the negative gradient of the total light field must be dominant inside the object. In other words, the way to realize an OPF translates to a problem of how to realize the dominance of the negative gradient of the total field inside the object.

**Fig. 1:**
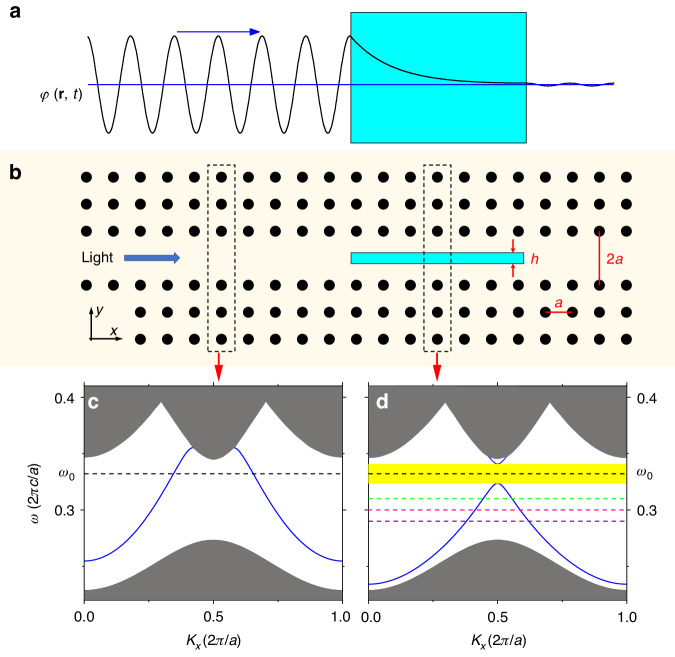
**a** Schematic diagram of a wave function decay inside a barrier. **b** Schematic diagram of a square lattice PC with a line-defect waveguide. The black circles represent the silicon cylinders forming a PC, while the cyan rectangle represents the object to be manipulated. **c** Dispersion curve of a guided mode in the PC waveguide without an object (blue line). The dispersion inside a perfect PC is shown with shaded grey areas. The operational frequency of *ω*_0_ = 0.332 × (2π*c*/*a*) is marked with a black dashed line. **d** Dispersion curve of a guided mode in the PC waveguide in the presence of an object. The spectral range indicated in yellow marks a band gap inside the waveguide. The green, pink, and purple dashed lines indicate the frequencies of *ω* = 0.31 × (2π*c*/*a*), 0.30 × (2π*c*/*a*), and 0.29 × (2π*c*/*a*), respectively, which are outside the band gap

In quantum mechanics, when a particle tunnels through a potential energy barrier with a height greater than the energy of the particle (*E*_particle_ < *V*), the wave function penetrates and exponentially decays into the barrier region (Fig. 1a). In optics, a similar effect is achieved with electromagnetic waves when they are incident on a PC and their frequencies are inside the band gap. To achieve a negative light field gradient inside the object, we introduce a line-defect waveguide in a two-dimensional PC (Fig. 1b). The employed square-lattice PC with a lattice constant *a* = 0.5 µm is composed of silicon cylinders (refractive index *n* = 3.47) with a diameter *d* = 0.4*a* and is submerged in water (*n*_w_ = 1.33). The manipulated object has a width of *h* = 0.2 µm (*n*_p_ = 2) and is positioned in the waveguide. The supercell technique was used to calculate the band gaps and guided modes of the waveguide with and without the manipulated object^43^. In Fig. 1b, the two schematic diagrams of the supercells, representing these two situations are marked with dashed rectangular boxes.

Without the object in the waveguide, the guided mode lies in the band gap of a perfect PC in a broad spectral range (Fig. 1c). Therefore, light at a frequency of *ω*_0_ can be guided along the waveguide. When an object (for example, of a rectangular shape) is placed in the center of the waveguide (Fig. 1b), a band gap appears for the guided modes at the location of the object (marked in yellow in Fig. 1d). In this case, the light with the frequency of *ω*_0_ cannot propagate in the waveguide region occupied by the object. Although the object considered here is of rectangular shape in order to elucidate the OPF mechanism, any shape of an object can be used as long as it opens the band gap.

**Fig. 2:**
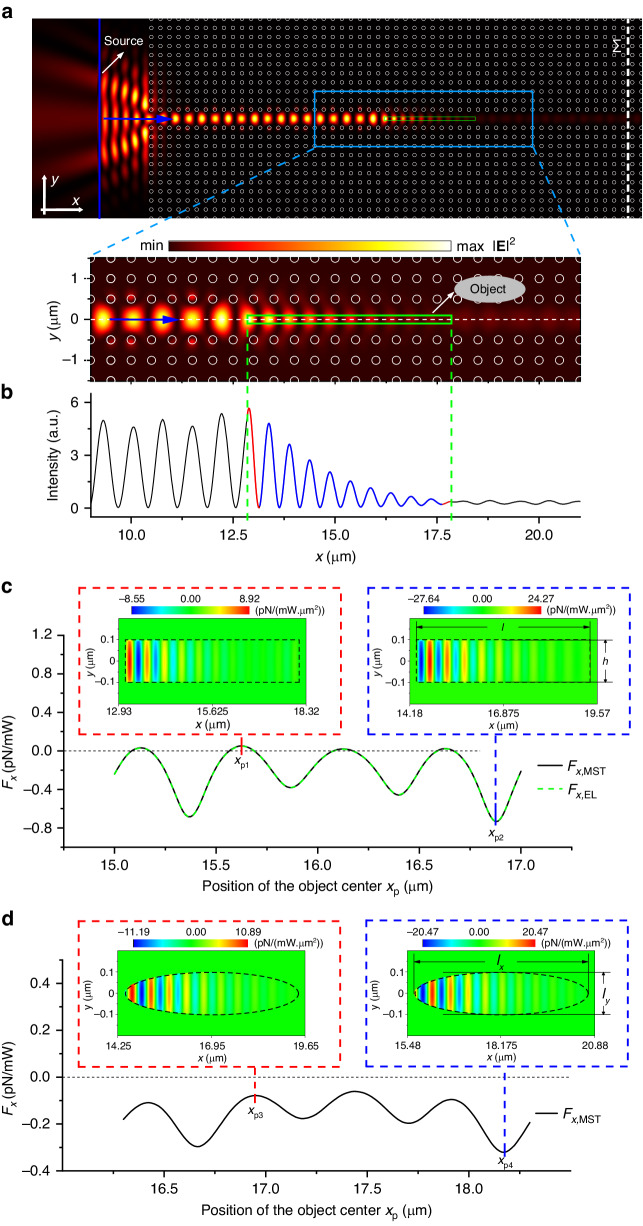
**a** Top view of the PC and the distribution of the light intensity |**E** | ^2^ after interaction of the incident fundamental TM mode of the waveguide with the rectangular manipulated object outlined by the green rectangle, with its center located at *x*_p_ = 15.35 µm. White circles indicate the PC silicon cylinders and the direction of incident waveguide mode is shown by the blue arrow. The white dashed line indicates a reference plane Σ for calculating reflectivity. **b** Field intensity distribution along *x*-axis at *y* = 0. **c** The force *F*_*x*_ acting on the rectangular object as a function of its position *x*_p_ (calculated with the E-L (green dashed line) and the MST (black solid line) approaches). Insets show the force density distribution of *F*_*x*_ inside the object at *x*_p1_ = 15.625 µm and *x*_p2_ = 16.875 µm, respectively. **d** The force *F*_*x*_ acting on an elliptical object as a function of its central position *x*_p_ (calculated with MST approach). Insets show the force density distribution of *F*_*x*_ inside the object at *x*_p3_ = 16.95 µm and *x*_p4_ = 18.175 µm, respectively. Force densities in all insets are calculated using the E-L approach

We consider an object with a length sufficient to open a gap in the dispersion (for example ∼ 3.3*λ*_0_ ≈ 5 µm). As shown in Fig. 2a, b, the TM polarized light with the frequency of *ω*_0_ propagating along the *x*-direction excites a fundamental TM guided mode in the line-defect PC waveguide. When it encounters the manipulated object, the electric field experiences attenuation, particularly inside the object. This attenuation is due to the bandgap opened in the waveguide where the object is present (Fig. 1). The time-averaged total force exerted upon the manipulated object can be obtained from Eq. (3), or, alternatively, by integrating the Maxwell’s stress tensor (MST) over the object surface^32^.

4 $$\begin{array}{l}\langle {{\bf{F}}}_{{\rm{MST}}}\rangle ={\int }_{s}\langle \overleftrightarrow{{\bf{T}}}\rangle \cdot d{\bf{s}}\,\,{\rm{with}}\\\quad\,\, \langle \overleftrightarrow{{\bf{T}}}\rangle =\frac{1}{2}\Re [{\bf{D}}\otimes {{\bf{E}}}^{\ast }+{\bf{H}}\otimes {{\bf{B}}}^{\ast }-\frac{1}{2}\overleftrightarrow{{\bf{I}}}({\bf{E}}\cdot {{\bf{D}}}^{\ast }+{\bf{H}}\cdot {{\bf{B}}}^{\ast })]\end{array}$$

Here, 〈·〉 indicates the time-average, ⊗ stands for the dyadic operation, and $\overleftrightarrow{{\bf{I}}}$ is the unit tensor. The dependence of the force acting on the object on the position of the object along the waveguide is presented in Fig. 2c. It can be seen that *F*_*x*,EL_ (the *x*-component of the total force) obtained from Eq. (3) coincides with *F*_*x*,MST_ obtained from Eq. (4), and the manipulated object is subjected to a pulling force in most of the object locations. This is because the envelope of the field intensity decays along the object, leading to an imbalance between the positive and negative field gradients. Insets in Fig. 2c show how the force density varies inside the object along its length following the negative and positive slopes in the field intensity, presented for the cases corresponding to the maximal and minimal values of the resulting integral force (see Section 1 of Supplementary information for more details).

Using the imbalance between the positive and negative slopes in the force density distribution resulting from the exponential decay of the fields inside the object for the frequencies corresponding to the bandgap, it is possible to realize the long-range OPF in all the positions along the waveguide by adjusting the shape of the object. As shown in Fig. 2d, the long-range OPF is achieved along the length of the waveguide if an elliptical object instead of a rectangular one is used. This is because the gradually narrowing profile of the ellipse eliminates the force at the rectangle corners and thus changes the ratio of positive and negative forces inside the object. The force density distribution for the ellipse center positioned at *x*_p3_ is shown in the left inset of Fig. 2d. The oscillations of the force density distribution are similar to those for a rectangular shape in the corresponding left inset of Fig. 2c, but due to the end tip geometry, the positive part of the force is cut out more than the negative force part which follows it. Thus, the total force upon an object changes from a small positive force for a rectangular shape to a negative force for an elliptical one. When an ellipsoid is located at *x*_p4_, the opposite effect is observed (cf. right insets in Fig. 2d, c). The magnitude of the total negative force becomes smaller compared to that for a rectangular object, but it still remains negative due to the very high-valued negative force for a rectangular object. This photonic band-gap-based OPF is of the same order of magnitude as that obtained by other methods^21^.

**Fig. 3:**
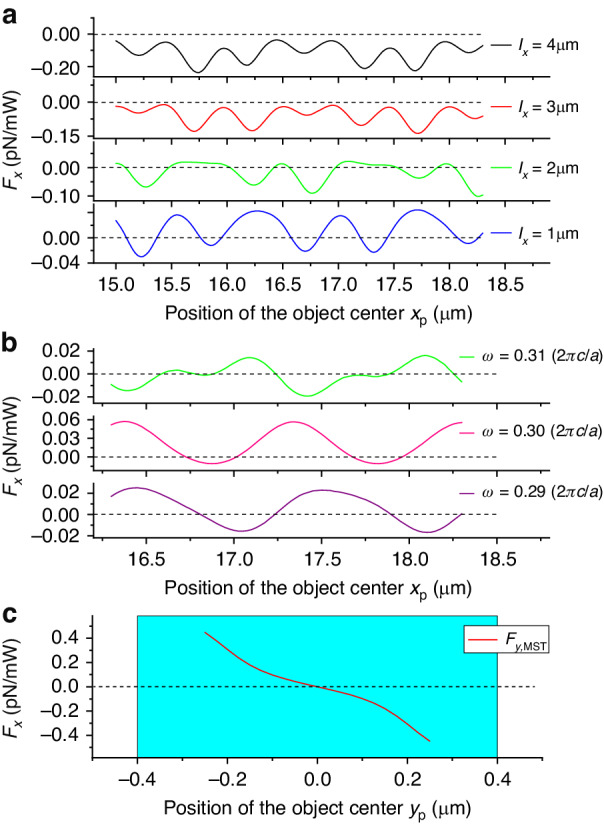
**a** The optical force acting on an elliptical object as a function of its position for various values of long axis *l*_*x*_, with a fixed *l*_*y*_ = 0.2 µm; the black dashed line marks *F*_*x*_ = 0. **b** The optical force acting on an elliptical object (*l*_*x*_ = 5 µm and *l*_*y*_ = 0.2 µm, same as in Fig. 2d) for various frequencies of the incident mode outside the band gap (shown in Fig. 1d). **c** The *y*-component of force, *F*_*y*_, as a function of the position of an elliptical object *y*_p_ (the dimensions of the ellipse are the same as in **b**). The *x*-coordinate of the center is fixed at *x*_p_ = 16.5 µm. The cyan rectangle marks the width of the waveguide. In this figure, the optical forces are all calculated using the MST approach

The band gap shown in Fig. 1d is calculated for an object which is infinitely long along *x*-direction. However, when the length of an object is reduced, the band gap effect weakens. In particular, as the long axis of the ellipse decreases, the OPF acting on the object diminishes until noticeable pushing forces appear (Fig. 3a). Similarly, when the frequency of the incident mode is outside the band gap, the light can pass through the object without attenuation, so long-range OPF cannot be achieved (Fig. 3b).

When an object is located off-axis, it is subjected to a force not only in the *x*-direction (*F*_*x*_) but also in the *y*-direction (*F*_*y*_). For the horizontal position of the center of the elliptical object fixed at *x*_p_ = 16.5 µm, the variation of *F*_*y*_ with change of *y*_p_ is shown in Fig. 3c. Analyzing the force curve, it can be seen that the position *y*_p_ = 0 is the stable equilibrium point in the *y*-direction. In other words, the object can be trapped on the central axis of the channel, which is very beneficial for the effective transportation of objects.

From the conservation law of the linear momentum, it follows that the backward scattering or reflection induces a pushing force on the object, which may counteract or even overcome the OPF. Therefore, a rational choice of the profile of light beam together with shape and material of the object needs to be implemented to eliminate the backward scattering which is crucial for obtaining the OPF^12,13^. The reflectivity can be calculated as *R* = 1 − *P*_with_/*P*_without_, where *P*_with_ and *P*_without_ are the values of the total power crossing the reference plane Σ, indicated in Fig. 2a, with and without an object inside the waveguide, respectively. This power *P* can be obtained as.

5 $$P=\frac{1}{2}{\int }_{\sum }({\mathbf{E}}\times {\mathbf{H}})\cdot d{\rm{s}}$$

In a typical case presented in Fig. 2a, the reflectivity is approximately 60 ~ 70%, depending on the object position. In a simplified model, we consider *N* photons propagating in the waveguide, each carrying a forward momentum *ħk*. Therefore, ∼0.7 *N* photons are reflected backward. Under an assumption that the reflection is caused only by an object, according to the conservation of the linear momentum, the object should obtain a forward momentum of 2 × 0.7*Nħk*, i.e. an object should be subjected to a pushing force. At the same time, Fig. 2d indicates that the object is actually subjected to a pulling force, which seems to mean that the law of conservation of the linear momentum is violated during the reflection process. It should be noted, however, that the band gap effect originates from the combined action of the object and the surrounding PC. Therefore, there are three objects participating in the momentum exchange: the photons, the manipulated object, and the lattice. In our case, the lattice absorbs an additional momentum and experiences a pushing force. If the manipulated object and the surrounding lattice are viewed as a complete system, then the total force acting upon them is a pushing force. Thus, although the reflectivity is very high, it does not violate the law of conservation of momentum (see Section 2 of Supplementary information for more details).

The proposed approach to achieve OPF using a photonic band gap is very flexible. For any object with particular characteristics, such as refractive index, shape, and size, a PC and a PC waveguide can be designed in order to achieve a required band gap, and consequently lead to OPF (see Section 3 of Supplementary information for more details).

**Fig. 4:**
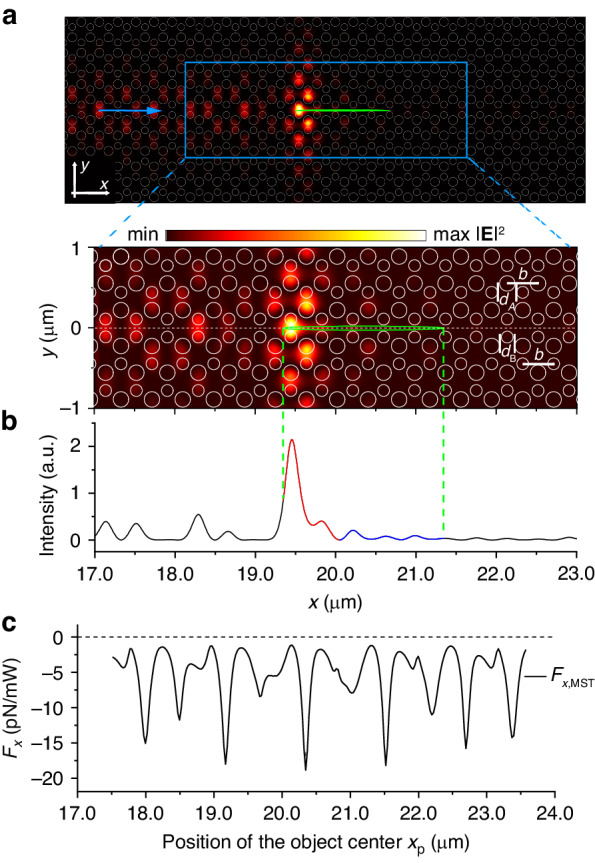
**a** OPF realized in a double-lattice PC (lattice constant *b* = 0.385 µm, *d*_A_ = 0.5*b*, *d*_B_ = 0.38*b*; a vertical mirror inversion of the design produces the waveguiding channel). This PC is composed of silicon cylinders (refractive index *n* = 3.47) and is submerged in water (*n*_w_ = 1.33). The upper figure shows the top view of the PC and distribution of the light intensity |**E** | ^2^ for the fundamental TM guided mode of the frequency of *ω*_1_ = 0.254 × (2π*c*/*b*) (within the band gap) interacting with an elliptic object with *l*_*x*_ = 2 µm and *l*_*y*_ = 0.05 µm. The white circles represent the silicon cylinders, the green ellipse represents the object, and the blue arrow shows the direction of the incident light. The lower figure shows an enlarged view of the area enclosed by a blue rectangle. **b** Distribution of light intensity along the line of *y* = 0. The vertical dashed green lines mark the edges of the manipulated elliptical object. **c** The force *F*_*x*_ acting on the object as a function of its position *x*_p_ (calculated with the MST approach)

Using the underlying physical principles, the magnitude of an OPF can be engineered by adjusting the light intensity at the front apex of an object. For example, a transverse optical resonance in the waveguide excited in the presence of an object can be used to enhance the light intensity at the object apex. A double-lattice PC was selected for this purpose (Fig. 4). The light intensity distribution in this case is shown in Figs. 4a, b, while the OPF as a function of an object position is presented in Fig. 4c. The strong resonance of a cavity produced by two smaller Si cylinders occurs at the front apex of the object, which significantly enhances the local field intensity. The enhancement consequently magnifies the optical field gradient, thus increasing the magnitude of the OPF (Fig. 4c). Compared to the OPF for the non-resonant case in Fig. 2d, the magnitude of the force is increased by a factor of ∼50. More details on this approach can be found in Sections 4 and 5 of Supplementary information.

An important consideration for the existence of an OPF is the effect of absorption. In general, due to inelastic interactions, the momentum of absorbed photons is transferred to the forward momentum of the object, resulting in an additional pushing force. Increased absorption reduces and eventually destroys the OPF^13,44,45^. It was previously shown that when the responsible for the absorption imaginary part of the refractive index of an object increases from 0 to 0.005i, the OPF completely degrades except for very limited object geometries^44^. In Refs. ^13^ and ^45^, typical imaginary parts of the refractive index for destroying the OPF were found to be less than 0.05i. For many materials, e.g. semiconductors (Ge^46^, InP^46^, etc.), the imaginary part of the refractive index is much greater than 0.05i in the visible spectral range (400 nm–800 nm), which brings a challenge to the universality in applications of the OPF. At the same, the OPF enhanced by the transverse resonance considered above is much less sensitive to absorption. In the case presented in Fig. 4, the optical force acting on the manipulated object remains negative when the complex refractive index of the object continuously changes from 3.47 + 0i to 3.47 + 3.27i (see Section 6 of Supplementary information for details). Thus, the proposed solution of using a transverse resonance to enhance the OPF creates a pathway for pulling objects with stronger absorption.

## Discussion

For practical applications, long-range OPFs stemming from the field gradients circumvent several challenges of the approaches based on scattering forces. Although ref. ^21^ provided motivation for the research on gradient-induced long-range optical forces, practical designs for gradient OPF realisation remained illusive until now. Indeed, while it was predicted that a self-collimation effect leading to a negative gradient of the light intensity inside an object might result in a pulling gradient force acting on an object^21^, it was not possible to ensure that the self-collimation effect will certainly produce the required negative gradient or that the gradient force will dominate in the total optical force. Therefore, seeking a universal theoretical approach for obtaining an OPF is of key importance for applications. To this end, we proposed a universal mechanism for generating the OPF based on the photonic band gap effect, which ensures that for any object a PC waveguide can be designed so that the object-induced band gap will inevitably lead to the field decay within the object, as it always happens when the band gap condition is satisfied^47,48^. Furethermore, using the implemented E-L formulation for an optical force, we established the relationship between the local light intensity gradient and the total force, thus offering a universal platform for designing the OPF and providing an insight into its origin.

By comparing the results of the E-L and the LO formulations for optical forces, we showed that not only the total forces are the same, but also the only non-zero force density components *f*_*x*_ are identical (see Section 7 of Supplementary information for the details). Similar conclusions were obtained in ref. ^31^. We confirmed that the total force calculated using the E-L formulation also coincides with the force calculated using the Maxwell’s stress tensor approach (Fig. 2c). This indicates that the E-L formulation is a reliable universal tool for calculating the optical forces, despite ongoing discussions on the electromagnetic force density inside matter.

In terms of feasibility of the experimental realization, the required photonic band-gap waveguides have been achieved experimentally in various types of PCs^49–59^, including two-dimensional PCs with a high aspect ratio of the cylinders^49–51^. They are well described by the two-dimensional PC model with an infinite cylinder length in the *z*-direction considered in our model and can be used to observe the OPF. A more practical method, however, would be to implement a two-dimensional PC slab sandwiched between two Bragg reflectors (multilayer dielectric structures) to confine the guided mode in *z*-direction. Such designs have been widely used in vertical-cavity surface-emitting lasers^52–56^. The Bragg reflectors can be interfaced with the PC slab using multiple technologies, e.g. direct etching^54,57^, wafer bonding^52,55^, and transfer-printing^56^. One can prepare a two-dimensional PC slab on the top of a pre-designed Bragg reflector through direct etching. Subsequently, another Bragg reflector can be connected to it through the wafer bonding technology (see Section 8 of Supplementary information for an extended discussion of a PC slab geometries and potential temperature effects). Alternatively, the localization of the mode in three dimensions can be achieved using line-defects in three-dimensional PCs. Full three-dimensional photonic band gap crystals have been fabricated with various approaches^58,59^. Thus, the OPF can be naturally anticipated to be observed experimentally in a three-dimensional PC as well. The demonstrated approach for the realization of the OPF can find applications in optical sorting^60^ and the realization of an optical conveyor for the transportation of small nano-objects^61^. As was discussed above, it is also robust against absorption in nano-objects and absorption in the PC can be designed to be minimal (Section 8 of Supplementary information).

The concept of the band gap design for optical waves has been extended to other types of waves, including acoustic waves and water waves. The method proposed here to obtain a pulling force through the band gap formation can be extended to acoustic and water waves. Taking water waves as an example, the band gap can be realized using two-dimensional periodic structures composed of vertical cylinders in a water tank^62^. Furthermore, on the surface of the liquid, the sign of the gradient force depends on whether the floating particle is hydrophilic or hydrophobic^63^. This pulling force on the water surface based on band gap effects may lead to useful applications, such as collecting crude oil particles from the ocean.

In summary, we have proposed and investigated a long-range OPF produced by a negative intensity gradient. The E-L formalism, clearly showing that the negative intensity gradient inside the manipulated objects produces a pulling force, was used both to calculate the OPF and to design optical structures for its enhancement. In particular, for an object placed in a line-defect waveguide of a PC, it was shown that at the photonic band gap conditions produced by the presence of the object, the imbalance between positive and negative intensity gradients present inside the object produces an OPF. The obtained results bring new insights on understanding of gradient optical forces, while the developed approach based on PC-based waveguides may find new applications in manipulation of nano-objects and can be extended to optofluidic and acoustic devices.

## Materials and methods

### Band gap calculations of PCs

The dispersions of the TM modes (electric field perpendicular to the *x-y* plane) presented in Fig. 1c, d were obtained using the plane-wave expansion method.

### Calculations of optical force

The light field distributions in the PC waveguide were simulated using the finite-difference time-domain (FDTD) method. The entire model is submerged in water and surrounded by perfectly matched layer (PML) boundary. Based on the obtained light field distribution, the optical forces **F**_EL_ and **F**_MST_ were calculated using Eqs. (3) and (4), respectively.

### Supplementary information

Supplementary information for Gradient-induced long range optical pulling force based on photonic band gap

## Data Availability

All the data supporting the findings of this study are presented in the text and Supplementary information and available from the corresponding authors upon reasonable request.
