# Supplementary material for: Gradient-induced long-range optical pulling force based on photonic band gap
Source: Light Sci Appl. 2024 Apr 24;13:93. doi: 10.1038/s41377-024-01452-y (PMC11039753; doi:10.1038/s41377-024-01452-y)
Supplement: Supplementary file 1 — Supplementary information for Gradient-induced long range optical pulling force based on photonic band gap [file 41377_2024_1452_MOESM1_ESM.docx]

Supplementary information for

**Gradient-induced long****‑range optical pulling force based on** **photonic band gap**

Wenlong Lu^1^, Alexey V. Krasavin^2^, Sheng Lan^1^, Anatoly V. Zayats^2*^, Qiaofeng Dai^1*^

1 Guangdong Basic Research Center of Excellence for Structure and Fundamental Interactions of Matter, Guangdong Provincial Key Laboratory of Nanophotonic Functional Materials and Devices, School of Information and Optoelectronic Science and Engineering, South China Normal University, Guangzhou, 510006, China.

2 Department of Physics and London Centre for Nanotechnology, King’s College London, London, WC2R 2LS, UK.

Anatoly V. Zayats

Email: a.zayats@kcl.ac.uk

Qiaofeng Dai

Email: daiqf@scnu.edu.cn

* Corresponding author.

1. **Analysis of the force density distribution**
   1. Positive force acting on a rectangular object

The parameters of a rectangular object are as follows: the refractive index *n*_p_ = 2, the width *h*= 0.2 μm and the length *l*= 5 μm. The center of the object is located at *x*_p1_ = 15.625 μm. As can be seen from the field intensity distribution shown in Fig. S1(b), an incomplete intensity peak (marked with the red line in Fig. S1(b)) appears only near the right end of the object, while complete intensity peaks (marked with the blue line in Fig. S1(b)) appear in the rest of the object. In accordance with the intensity peak positions, the object is divided into several intervals along the *x‑*axis. The contribution of each interval to the total force is shown in Fig. S1(e). The total force acting on the object is calculated to be *F_x_*_,total_ = 0.024 pN·mW^-1^, which mainly originates from a positive gradient force occurring in the rightmost interval.

- 1. Large negative force acting on a rectangular object

A rectangular object with the same parameters as in Fig. S1 is considered, but with the center shifted to *x*_p2_ = 16.875 μm. From the field intensity distribution shown in Fig. S2(b), one can see that the incomplete intensity peaks (marked with the red line in Fig. S2(b)) appear near both ends of the object, while the complete intensity peaks (marked with the blue line in Fig. S2(b)) appear in the rest of the object. In accordance with the intensity peak positions, the object is divided into several intervals along the *x‑*axis. The contribution of each interval to the total force is shown in Fig. S2(e). The leftmost interval produces a negative gradient force, while the rightmost interval produces a positive gradient force. Due to the decaying nature of the envelope of the field intensity inside the object, the magnitude of the negative force is larger than that of the positive force. The total force acting on the object is calculated to be *F_x_*_,total_ = −0.67 pN·mW^-1^.


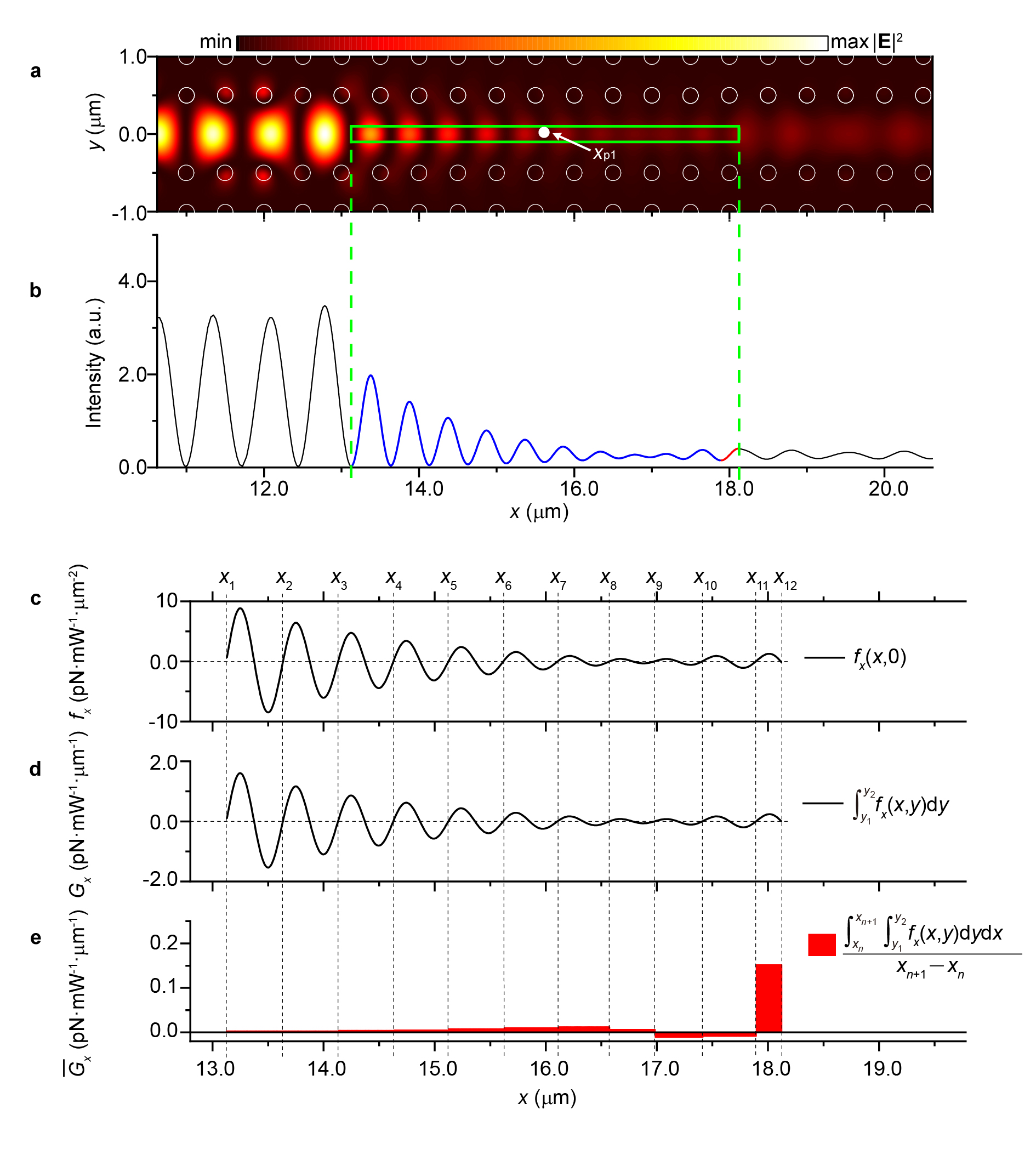


Fig. S1. (a) 2D field intensity distribution in the photonic crystal (PC) waveguide and inside the manipulated object. The object profile is outlined by the green rectangle. (b) The field intensity distribution along the *x‑*axis at *y*= 0. (c) The distribution of the *x* component of the optical force density inside the object, *f_x_*, along the *x‑*axis at *y*= 0. (d) The distribution of *G_x_*, the integral of *f_x_*(*x,y*) along the *y‑*direction (*y*_1_ and *y*_2_ mark the boundaries of the object), along the *x‑*axis. (e) The average value of *G_x_* for each spatial interval. The areas of the red rectangles indicate the magnitude of optical force density integrated over each interval.


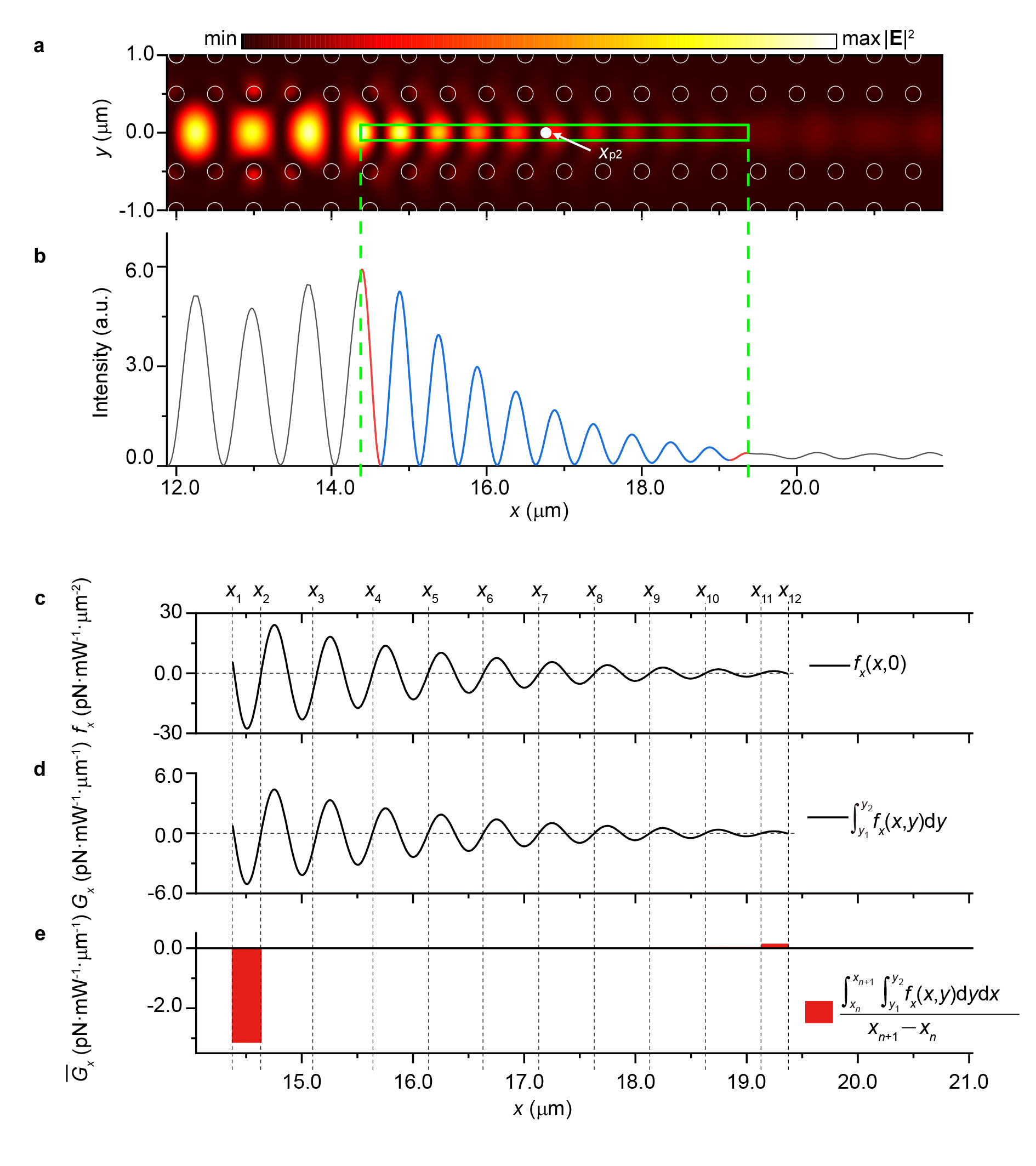


Fig. S2. (a) 2D field intensity distribution in the PC waveguide and inside the manipulated object. The object profile is outlined by the green rectangle. (b) The field intensity distribution along the *x‑*axis at *y*= 0. (c) The distribution of the *x* component of the optical force density inside the object, *f_x_*, along the *x‑*axis at *y*= 0. (d) The distribution of *G_x_* along the *x‑*axis. (e) The average value of *G_x_* for each spatial interval. The areas of the red rectangles indicate the magnitude of optical force density integrated over each interval.

**2. Pulling force analysis from the perspective of linear momentum**

The conservation of the linear momentum is discussed on the example of an elliptical object placed in a square*‑*lattice PC at *x*_p4_ = 18.175 μm. The forces acting on the object and lattice of the PC are presented in Fig. S3. Light propagates in the waveguide and is reflected when it encounters the band gap formed by the object and the surrounding lattice. Upon the reflection, both the object and the lattice participate in the linear momentum exchange with the photons. When the object is subjected to the optical pulling force (OPF), the lattice above and below the object is subjected to an optical pushing force. Considering the object and the lattice as a complete system, the total force on the latter is positive, which indicates the conservation of the linear momentum.


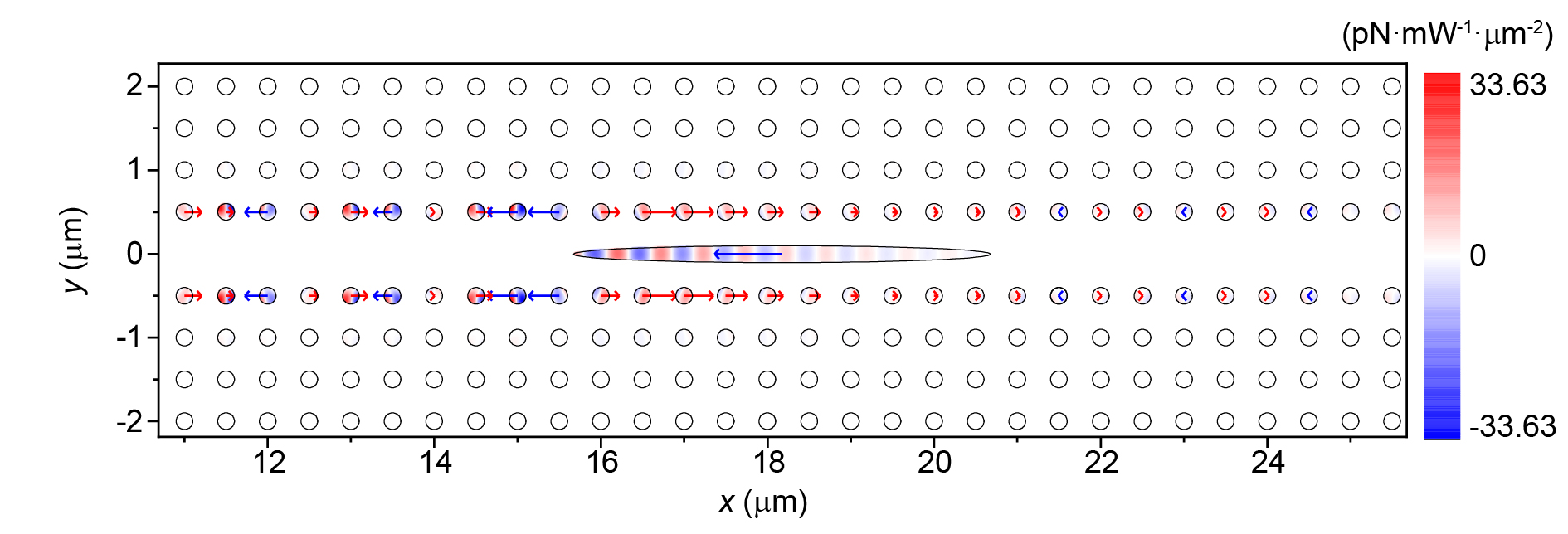


Fig. S3. The directional map of the optical forces (arrows) and the force density distribution (color map). Red arrows indicate positive (pushing) forces, and blue arrows indicate negative (pulling) forces. The length of the arrows is proportional to the magnitude of the force. An elliptical object (*n*_p_ = 2) has a long axis of *l_x_* = 5 μm and a short axis of *l_y_* = 0.2 μm. The force acting on the object is calculated to be *F_x_*_,total_ = −0.32 pN·mW^-1^.

**3. Influence of refractive index and size of object on OPF**

The influence of the refractive index and width of the object on the optical force was investigated for an object of a rectangular shape presented in Fig. 1(b). As the first step, the dependence of a band gap width on the object parameters was studied as shown in Fig. S4(a). Among various combinations, two cases were selected as characteristic examples, their band structures and OPFs are shown in Figs. S4(b)—S4(e). The results indicate that the developed approach provides a robust platform for achieving OPFs for objects with various refractive indexes and sizes.


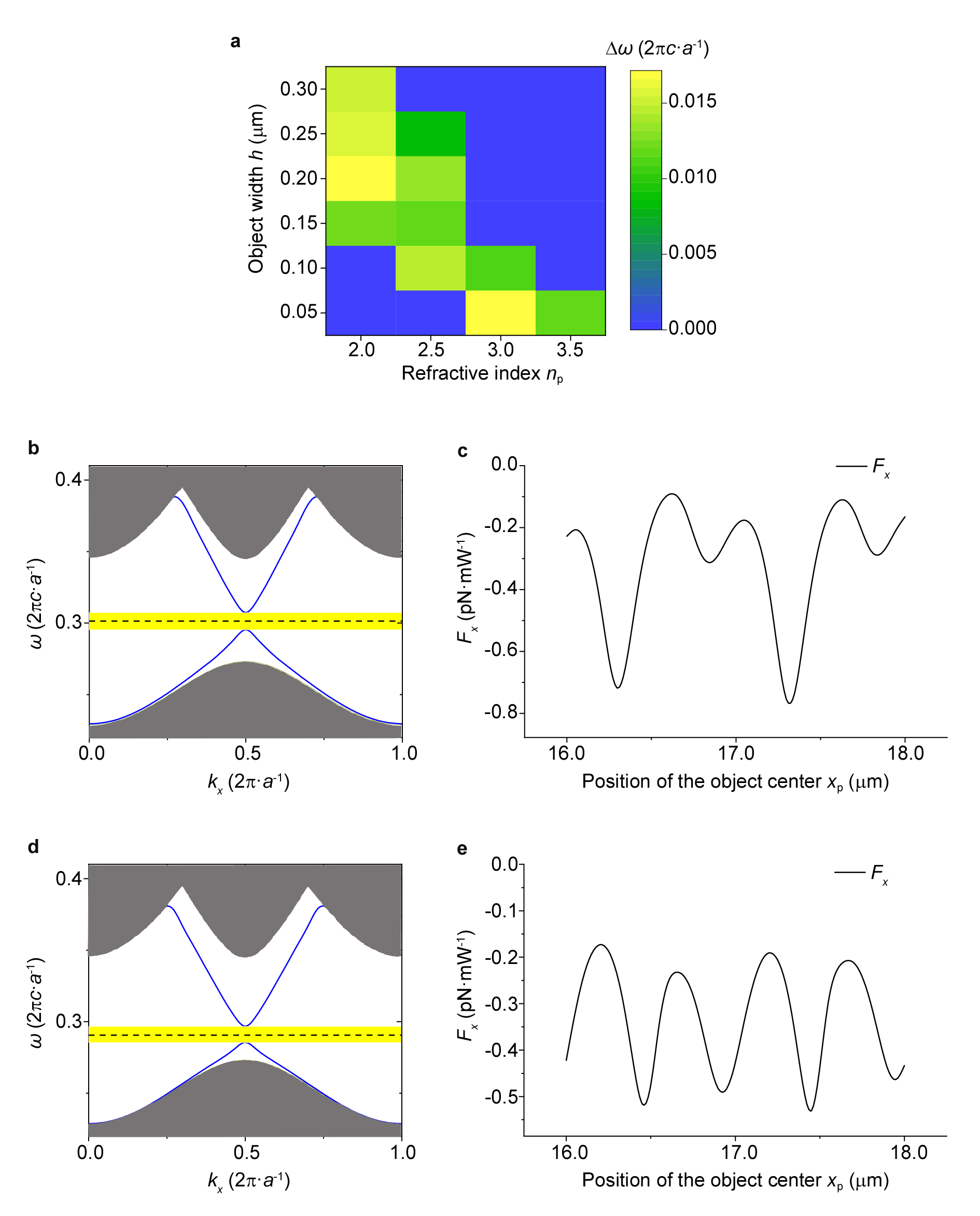


Fig. S4. (a) Parametric plot of a band gap width against the object refractive index and width. (b) Band structure of the PC (shaded in grey) and the dispersion of the guided mode (blue lines) in the region containing an object with refractive index *n*_p_ = 2.5 and width *h* = 0.15 μm. The spectral range indicated in yellow marks a band gap, and the black dashed line marks the central band gap frequency of 0.3013 × (2π*c*·*a*^-1^). (c) The force *F_x_* acting on the object as a function of the central position of the object *x*_p_. The parameters of the object are as follows: refractive index *n*_p_ = 2.5, major axis *l_x_* = 6.5 μm and minor axis *l_y_* = 0.15 μm, which correspond to the parameters used in (b). The frequency of the incident light is 0.3013 × (2π*c*·*a*^-1^). (d) Band structure of the PC and the dispersion of the guided mode in the region containing an object with refractive index *n*_p_ = 3 and width *h* = 0.1 μm. The spectral range indicated in yellow marks a band gap, and the black dashed line marks the central band gap frequency of 0.2906 × (2π*c*·*a*^-1^). (e) The force *F_x_* acting on an object as a function of the central position of the object *x*_p_. The parameters of the object are as follows: refractive index *n*_p_ = 3, major axis *l_x_* = 7 μm and minor axis *l_y_* = 0.1 μm, which correspond to the parameters used in (d). The frequency of the incident light is 0.2906 × (2π*c*·*a*^-1^).

**4. Band gap in a double‑lattice PC**

We consider a PC composed of two kinds of cylinders with different diameters, arranged in hexagonal lattices shifted with respect to each other. Such structure is mirrored with respect to *y* = 0 to form a waveguide (Fig. S5, for clarity one side of the waveguide is shown in blue and the other in red). Fig. S5(a) presents the dispersion of a guided mode and the PC band structure. Light with a frequency *ω*_1_ = 0.254 × (2π*c*·*b*^-1^) can propagate along the waveguide. When an object with a width *h*= 50 nm and a refractive index *n*_p_ = 3.47 is placed in the waveguide, it opens a band gap (marked with a yellow rectangle in Fig. S5(b)). Thus, the guided mode cannot propagate in this region, and decays, producing an OPF, as discussed in the main text.


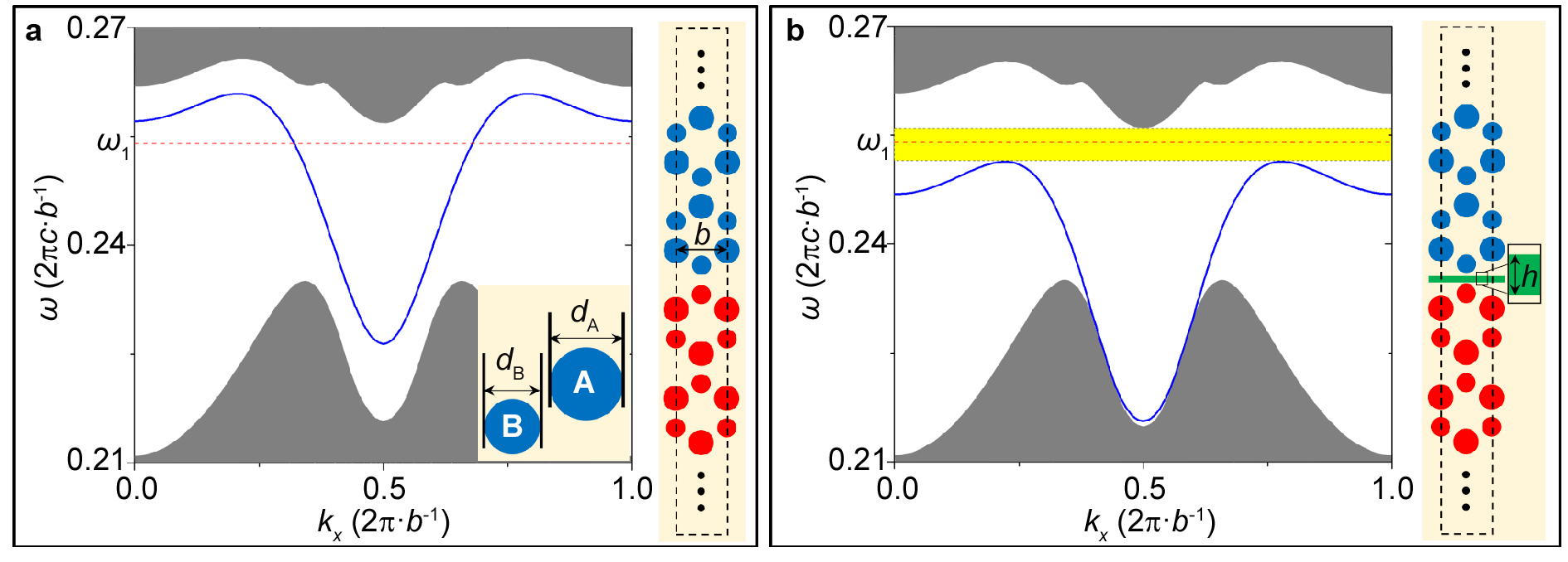


Fig. S5. Band structure of a double‑lattice PC in the waveguide direction and dispersions of the guided modes (a) without and (b) with the manipulated object present in the waveguide. The refractive index of the cylinders is *n*_p_ = 3.47, the lattice constant is *b* = 0.385 μm, the diameter of the large cylinders is *d*_A_ = 0.5*b*, the diameter of the small cylinders is *d*_B_ = 0.38*b*, the refractive index of the background media (water) is *n*_w_ = 1.33, the object is shown in green. The spectral range indicated in yellow marks a band gap.

**5.** **Resonance enhancement of OPF**

As can be seen in Fig. 4, the guided mode of the double‑lattice waveguide incident on an object experiences a resonant field enhancement at the object end. When calculating the band structure of the supercell along the *k_y_* direction of the Brillouin zone, one can obtain a transverse resonance mode in the band gap of the perfect PC. The frequency of the transverse resonance depends on the object width (Fig. S6(a)), thus for any object width *h*_0_, there is a resonant frequency *ω*_1_, which results in the significant field enhancement (Fig. S6(b)). Particularly, the resonant enhancement occurs near the position where the effective width of the elliptical object is equal to *h*_0_ = 18 nm (Fig. S6(a)). Such enhancement greatly increases the gradient of the intensity at the left end of the object.

For the square‑array waveguide presented in Fig. 1, the situation is quite different (Fig. S6(c)). Since the frequency range around *ω*_0_ = 0.332 × (2π*c*·*a*^-1^), which is used to achieve the OPF, has no intersection with the transverse resonance spectral curve, no resonance can be excited.

In the above two cases, the transverse resonance frequency decreases as the object width *h* increases. In order to excite the transverse resonance for an object with a given width *h* = *h*_0_, the incident light frequency should be lower than the transverse resonance frequency at *h* = 0. Meanwhile, for a PC waveguide along the *x*-direction, the guided mode frequency at *k_x_* = 0 (in the *x*-direction) always coincides with the corresponding transverse resonance frequency (in the *y*-direction) for any given width of an object. This requires that the selected incident light frequency should also be lower than the guided mode frequency at *k_x_* = 0 of the waveguide without the object (i.e. *h* = 0). Therefore, as shown in Fig. S5(a), the guided mode dispersion curve with a higher frequency at *k_x_* = 0 and a lower frequency at *k_x_* = 0.5 × (2π·*b*^-1^) can meet the requirement. Such a guided mode profile can be obtained by optimizing the lattice symmetry, refractive index, filling ratio of the PC, and the width of the waveguide.


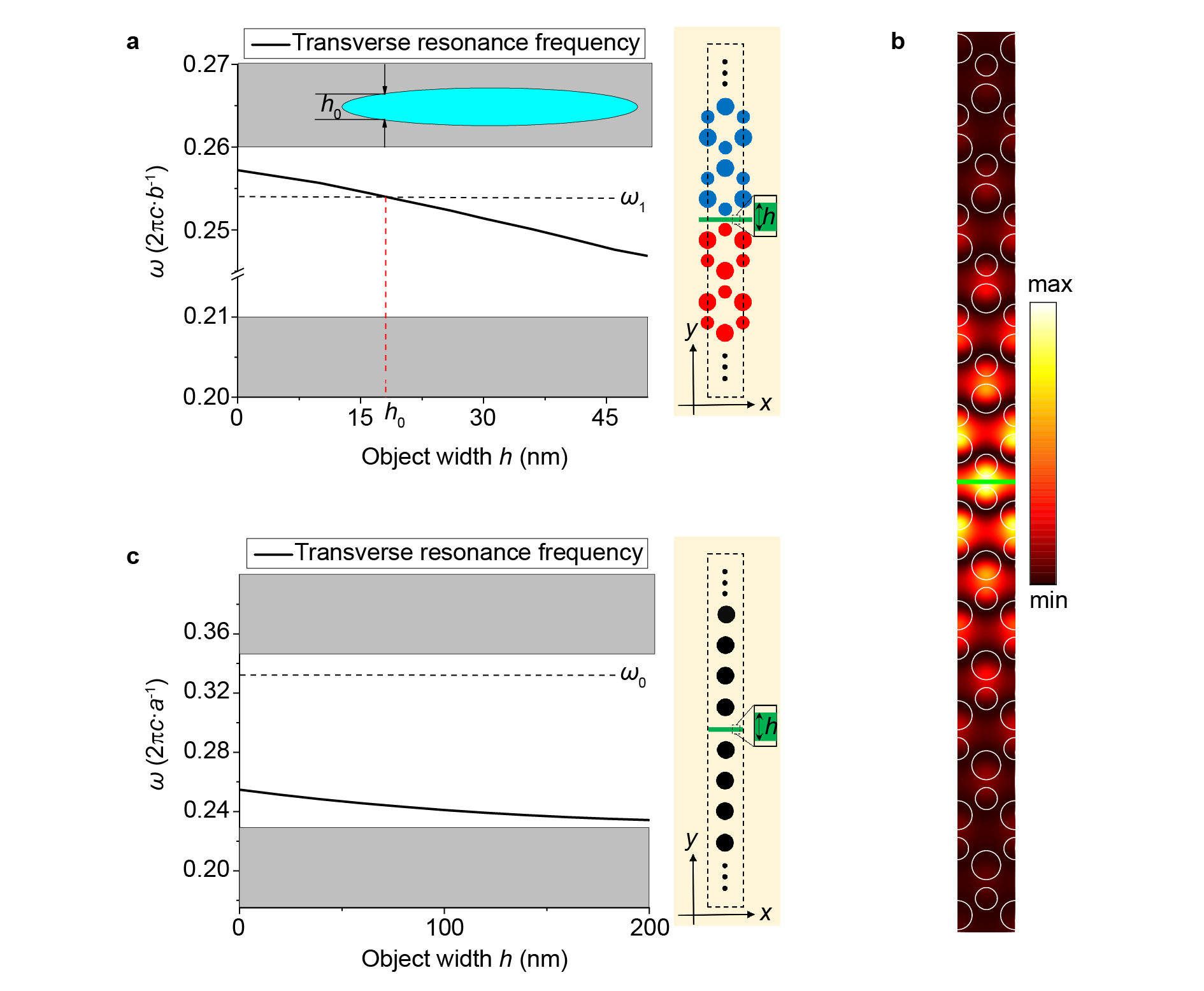


Fig. S6. (a) The dependence of the transverse resonance frequency on the object width for the double*‑*lattice PC. (b) The field intensity distribution for a manipulated object with a width *h*_0_ = 18 nm excited by the guided mode with a frequency *ω*_1_ = 0.254 × (2π*c*·*b*^-1^). (c) The dependence of the transverse resonance frequency on the object width for a square*‑*array PC.

**6. Influence of object absorption on OPF**

Considering absorption, the complex refractive index of a manipulated object is $\tilde{n}=n_{p}+{i\kappa}_{p}$, where *n*_p_ and *κ*_p_ are the refractive index and extinction coefficient, respectively. For the case of resonance manipulation in the double‑lattice waveguide presented in Fig. 4, the force remains negative when *κ*_p_ increases from 0 to 3.27 (Fig. S7(a)). For the comparison, the case of non-resonance manipulation in the square‑array PC waveguide in Fig. 2(d) was also studied, and the OPF is destroyed when *κ*_p_ is greater than only 0.028 (Fig. S7(b)).


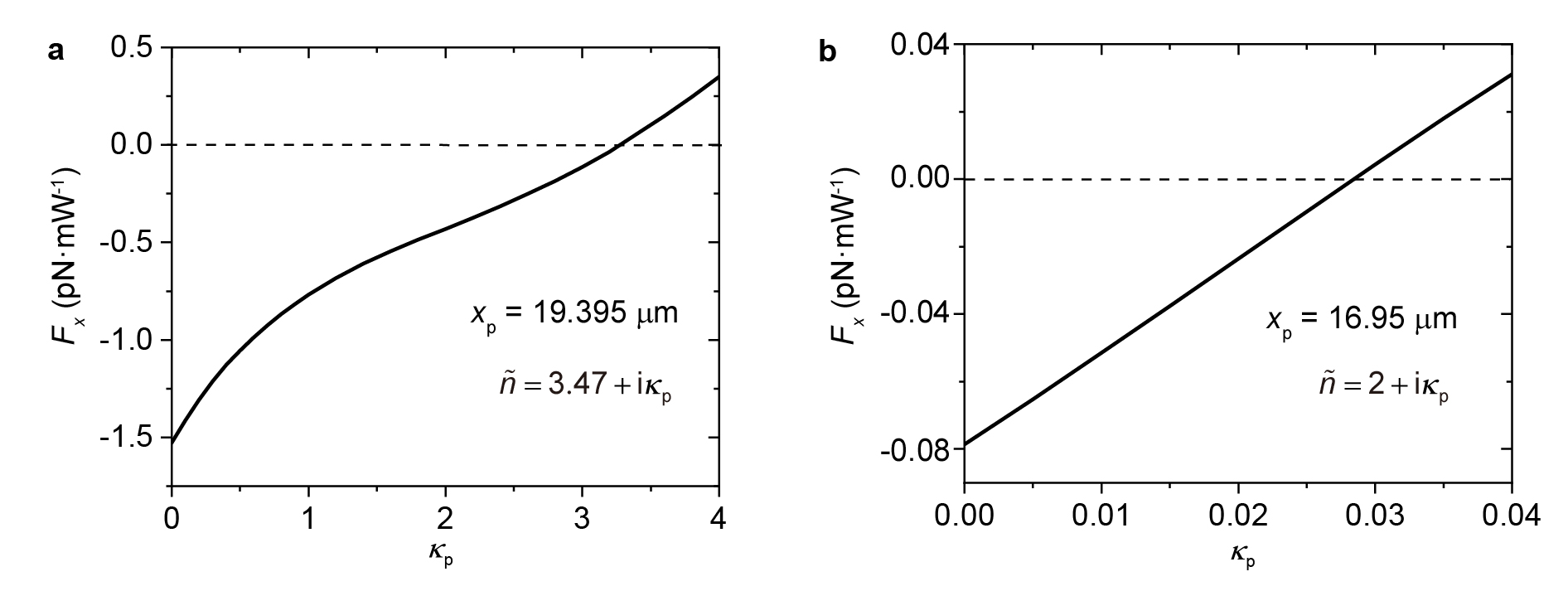


Fig. S7. The optical force as a function of the extinction coefficient of an object for (a) a double-lattice PC waveguide and (b) a square-array PC waveguide. The center of an object is located at *x*_p_ and $\tilde{n}$ denotes the complex refractive index of an object.

**7.** **Force density** **distributions derived from two formulations**

The force density distributions calculated using the Einstein-Laub and Lorentz formulations discussed in the main text are shown in Fig. S8. The force density in the Lorentz formulation is given by [1]:

$$\mathbf{f}_{\mathrm{LO}}= \left( \rho_{\mathrm{free}}-\nabla\cdot\mathbf{P} \right)\mathbf{E}+ \left( \mathbf{J}_{\mathrm{free}}+\frac{\partial\mathbf{P}}{\partial t}+\mu_{0}^{-1}\nabla\times\mathbf{M} \right) \times\mathbf{B}$$

while its counterpart in the Einstein-Laub formulation is given by Eq. 2 of the main text. It can be seen that the distributions derived from the two formulations agree very well, leading to the same total force of −0.194 pN·mW^-1^.


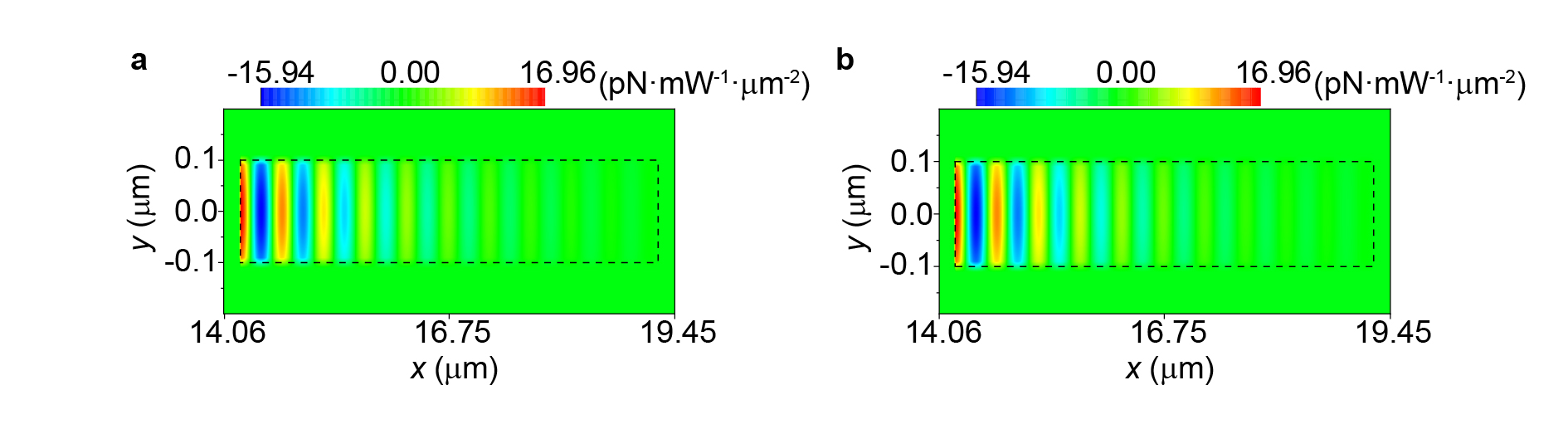


Fig. S8. The spatial distribution of the force density *f_x_* inside the object calculated using (a) the Einstein-Laub formulation and (b) the Lorentz formulation. The object with parameters as in Fig. 2(c) and the center located at *x*_p_ = 16.75 μm is selected as an example for the comparison.

**8. Temperature effects on OPF in PC slab**

The issues important for the experimental realization of OPF in the proposed PC configuration are related to absorption of the object, which was discussed above in Supplementary Section 6, and thermal effects associated with the absorption of the PC slab. Using self-consistent coupled modelling of electromagnetic and thermal phenomena in the finite-element formulation (COMSOL Multiphysics software), we calculated the force and the corresponding rise of the temperature in one of the most temperature-affected experimental configurations of a 2D PC slab sandwiched between two gold films (Fig. S9). The associated absorption of light in this system brings about two problems: (i) weakened light intensity will reduce the OPF and (ii) the increased temperature will disturb the OPF due to the Brownian motion.


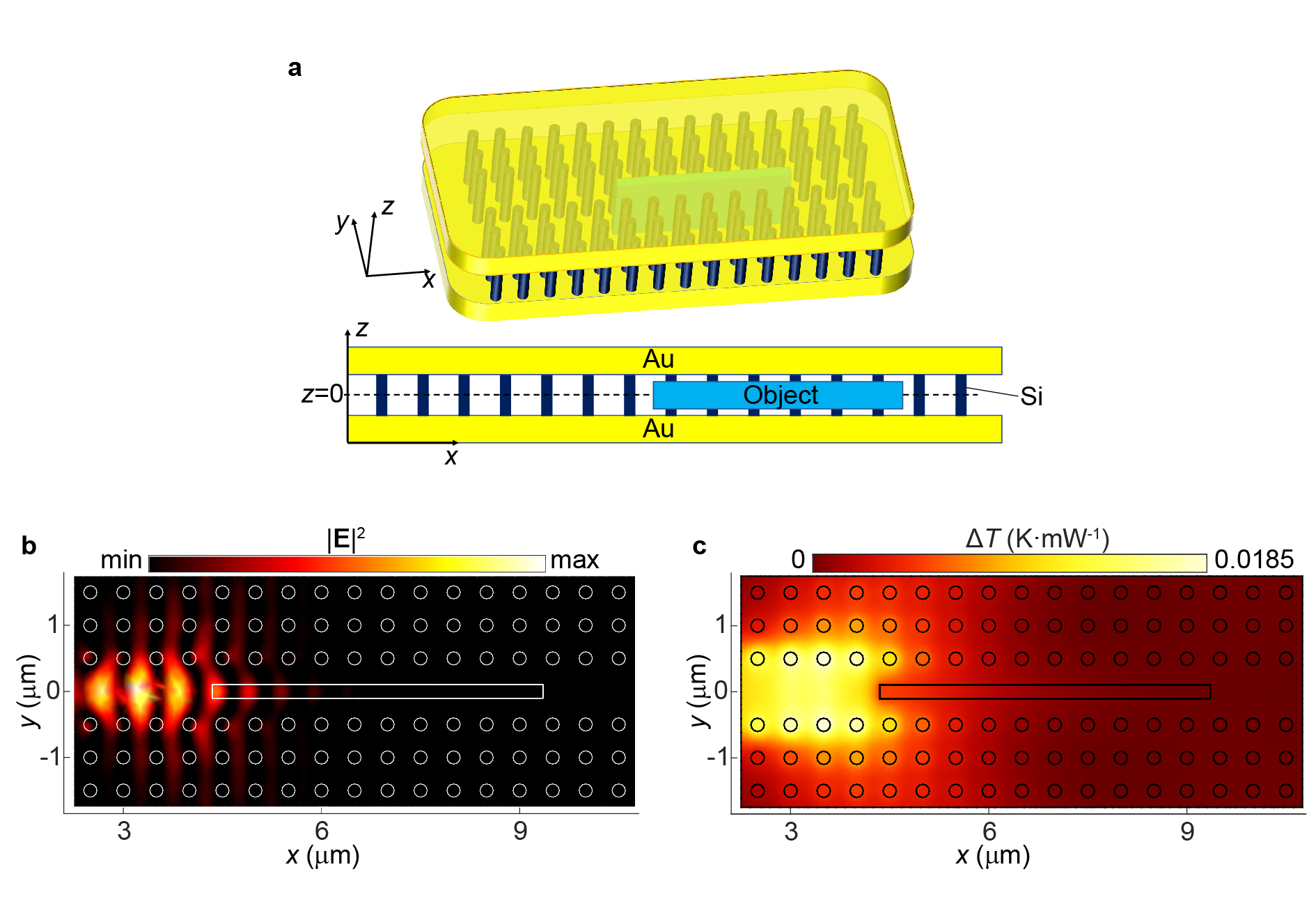


Fig. S9. (a) Schematic diagram of the implemented 3D model of a PC waveguide sandwiched between two gold films and its cross-sectional view. The PC slab has a thickness of 500 nm, a lattice constant of *a* = 500 nm, and each silicon cylinder has a radius of 0.2*a*. The object with a refractive index of *n*_p_ = 2 has a square geometry with a height of 400 nm, length of 5000 nm, and width of 200 nm. The refractive index of the background media (water) is set to be *n*_w_ = 1.33. The object is placed at the central axis of the waveguide, with its center at *x*_p_ = 6.85 μm, *y*_p_ = 0 μm and *z*_p_ = 0 μm. Simulated (b) optical intensity and (c) temperature rise distributions in the *z* = 0 plane. Circles represent silicon cylinders, while rectangular blocks mark the manipulated object.

As shown in Fig. S9, the optical intensity distribution inside the object in the *x-y* plane at *z* = 0 shows visible attenuation, which is very similar to that appearing in the 2D PC (Fig. 2(a) of the main text). The total force exerted on the object along the *x*-direction in this case amounts to *F_x_* = − 0.46 pN·mW^-1^, while forces acting along *y* and *z*-directions are *F_y_* = *F_z_* = 0. Furthermore, it was found that the thermal impact remains minimal throughout the system with the maximal temperature rise Δ*T* = 0.0185 K·mW^-1^. Therefore, at the considered sample position, the OPF acting on the object does not significantly decrease, compared to the lossless 2D case considered in the manuscript, and the temperature of the system does not increase to the levels at which Brownian motion and dynamics of the object might be significantly affected. In our design, we propose to use Bragg reflectors instead of metal films for confining the waveguided mode in *z*-direction, which are essentially non-absorptive and do not cause temperature-related problems.

**Supplementary Information References**

1. M. Mansuripur, A. R. Zakharian, and E. M. Wright, Electromagnetic-force distribution inside matter, Physical Review A **88**, 023826 (2013).
